# MicroRNA-153-5p promotes the proliferation and metastasis of renal cell carcinoma via direct targeting of AGO1

**DOI:** 10.1038/s41419-020-03306-y

**Published:** 2021-01-04

**Authors:** Zeyan Li, Shuo Zhao, Shiqin Zhu, Yidong Fan

**Affiliations:** 1 Department of Urology, Qilu Hospital, Cheeloo College of Medicine, Shandong University, Jinan, Shandong 250012 China; 2 Laboratory of Basic Medical Sciences, Qilu Hospital of Shandong University, Jinan, Shandong 250012 China; 3 Center for Reproductive Medicine, Cheeloo College of Medicine, Shandong University, Jinan, Shandong 250012 China

**Keywords:** Cancer screening, miRNAs

## Abstract

MicroRNAs (miRNAs) have been demonstrated to affect the biological processes of cancers and showed great potential for prognostic biomarkers. In this study, we screened differentially expressed miRNAs in ccRCC based on three dimensions of metastasis, prognosis, and differential expression compared to normal tissue using bioinformatics algorithms. MiR-153-5p was identified as a candidate miRNA to promote ccRCC occurrence and progression. Clinically, we found that miR-153-5p was significantly upregulated and related to unfavorable clinical features in ccRCC. Besides, miR-153-5p served as an independent prognostic biomarker. Functionally, miR-153-5p depletion remarkably inhibited the proliferation and metastasis of ccRCC via the phosphatidylinositol 3-kinase (PI3K)/Akt signaling. Furthermore, AGO1 was proved to be a direct target of miR-153-5p. AGO1 is associated with favorable clinical features and exhibited independent prognostic value in ccRCC. Besides, we observed that AGO1 knockdown significantly promoted tumor proliferation and metastasis. Downregulation of AGO1 partly abolished the oncogenic effects of miR-153-5p knockdown. Furthermore, miR-153-5p combined with AGO1 showed more robust prognostic significance in ccRCC. In conclusion, we found that the newly identified miR-153-5p/AGO1 axis was responsible for tumor occurrence and progression via PI3K/Akt signaling, which may therefore provide promising therapeutic targets and prognostic biomarkers for patients with ccRCC.

## Introduction

Renal cell carcinoma (RCC), as the third most common urological tumor, accounts for ~90% of kidney cancers and 4% of all adult malignancies^1^. Clear cell RCC (ccRCC) is the most common RCC subtype and rarely leads to symptoms in its early stages. However, metastatic diseases in the advanced stage instead of primary tumor impair patients’ lives by decreasing the chance of 5-year survival rate from 69.4% to 10%^2,3^. Surgical resection is the main curative therapy for ccRCC due to its resistance to chemoradiotherapy, while 30% of patients will still experience recurrence or metastasis after nephrectomy^4^. As ccRCC is highly heterogeneous and intricate in molecular pathogenesis, the underlying mechanisms of initiation and metastasis remain largely elusive. Thus, exploring new clinical biomarkers and therapeutic targets is urgently required for patients with ccRCC.

MicroRNAs (miRNAs), which are small noncoding regulatory RNAs, contribute to the regulation of multiple mechanisms regarding tumorigenesis and metastasis^5^. miRNAs function as tumor suppressors or oncomiRNAs through downregulating the transcription of cancer-related genes by binding directly to the target sites in its 3′-untranslated regions (3′-UTR)^6,7^. Recent studies have reported that several miRNAs, such as miR-543, miR-223-3p, and miR-200b, were dysregulated and showed prognostic significance in kidney tumors^8–10^. MiR-153-5p, generated from the 5′-arm of precursor miR-153, was found to be implicated in the pathological processes of cancer. A recent study has illustrated that miR-153-5p could abolish circPAN3-induced promotion of chemo-resistance in acute myeloid leukemia^11^. Interestingly, as the 3′-arm of precursor miR-153, miR-153-3p was reported to be downregulated and function as a tumor suppressor via targeting ZEB2 and Uc.416 + A in ccRCC^12,13^. However, given the complicated function and regulation of miRNA, the definite biological role of miR-153-5p underlying ccRCC remains to be fully investigated.

In our study, we systematically screened metastasis-related, prognosis-related, and differentially expressed miRNA in ccRCC using bioinformatics algorithms. MiR-153-5p was identified as overexpressed in ccRCC and closely related to poor prognosis and metastasis. We first demonstrated the oncogenic role of miR-153-5p and its underlying molecular mechanism in ccRCC. Moreover, we initially verified argonaute (AGO) RNA-induced silencing complex (RISC) catalytic component 1 (AGO1) as the direct target of miR-153-5p and the function of AGO1 as a tumor suppressor in ccRCC. According to our findings, we proposed that miR-153-5p combined with AGO1 may be a promising clinical prognostic assessment tool and therapeutic target.

## Results

### MiR-153-5p was identified as a candidate miRNA to promote tumor progression in patients with ccRCC

**Fig. 1:**
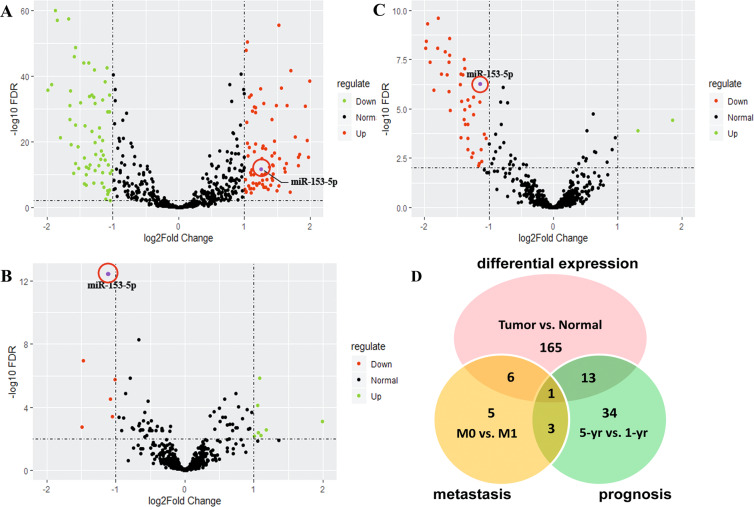
MiR-153-5p was identified as a candidate miRNA to promote tumor progression in patients with ccRCC. **A**–**C** Volcano plots depicting differentially expressed miRNAs between ccRCC and normal tissue **A**, between M1 and M0 **B**, and between <1-year survival (1-yr) and >5-year survival (5-yr) **C**. **D** Schematic illustration displayed the overlapping of miRNAs by three volcano plots.

To explore the ccRCC progression-related miRNA, we performed the differential expression analyses of miRNA using The Cancer Genome Atlas (TCGA) database through bioinformatics algorithms. The analyses were based on the three dimensions of metastasis, prognosis, and differential expression (vs. normal tissue) in ccRCC. For the differential analyses of metastasis, we divided the ccRCC patients into metastasis (M1) group and non-metastasis (M0) group, whereas for that of prognosis, the patients were allocated to <1-year survival group and >5-year survival (5-yr) group. Volcano plots showed that 185 miRNAs differentially expressed for differential expression groups (Fig. 1A), 15 miRNAs for metastasis groups (Fig. 1B), and 51 miRNAs for prognosis groups (Fig. 1C) with the filter of 2-folds. Among all the differentially expressed miRNAs, miR-153-5p occurred simultaneously in three volcano plots (Fig. 1D). Results indicated that miR-153-5p was significantly overexpressed in ccRCC tissue and downregulated in M0 groups as well as in the 5-yr group. Thus, we identified miR-153-5p as a candidate miRNA promoting ccRCC progression.

### MiR-153-5p is related to unfavorable clinical features and serves as an independent prognostic biomarker in ccRCC

**Fig. 2:**
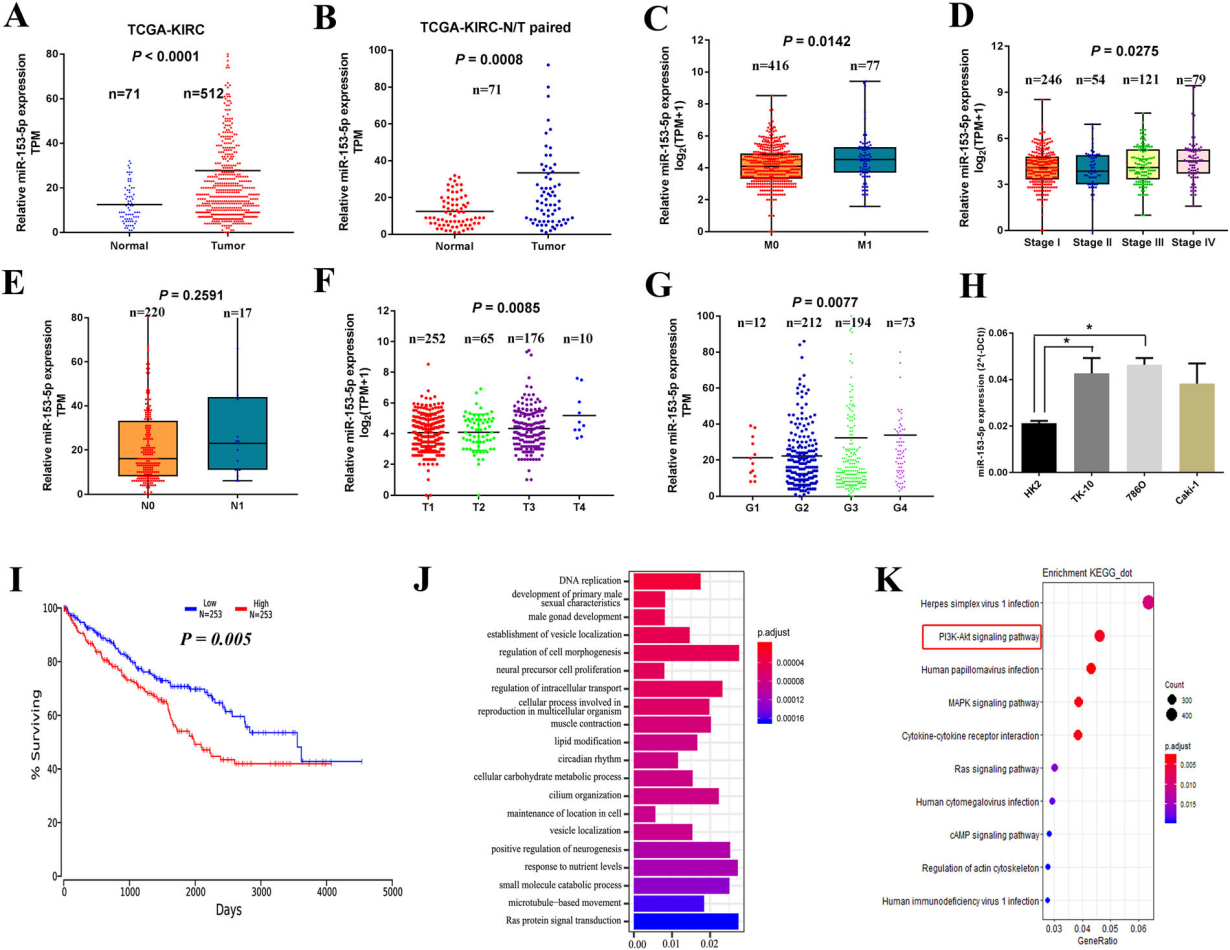
MiR-153-5p is aberrantly overexpressed in ccRCC and related to unfavorable clinical features and prognosis. **A**, **B** Relative expression of miR-153-5p in tumor-normal tissues (**A**) and paired tumor-normal tissues (**B**) of TCGA. Horizontal lines represent the mean value. **C**–**G** Correlation of miR-153-5p with clinicopathological characteristics, including metastasis (**C**), TNM stage (**D**), lymph node metastasis (**E**), T stage (**F**), and histologic grade (**G**). **H** The expression level of miR-153-5p in ccRCC cell lines and human renal tubular epithelial cells. Horizontal lines in **F**, **G** represent the mean value. Horizontal lines in the boxes in **C**–**E** indicated the median value. Boxes span the interquartile range. Whiskers extend from the minimum to the maximum. The values in **H** are presented as mean ± SEM. **I** Kaplan–Meier analysis of the correlation between miR-153-5p and overall survival in ccRCC. **J**, **K** Gene Ontology (GO) enrichment analysis (**J**) and Kyoto Encyclopedia of Genes Genomes (KEGG) pathways enrichment analysis (**K**) for the expression of miR-153-5p. All experiments were conducted at least three independent times. *P < 0.05.

We analyzed the correlation of miR-153-5p with clinicopathological characteristics and overall survival in ccRCC. As shown in Fig. 2, the expression levels of miR-153-5p were significantly increased in ccRCC than those in normal tissue (Fig. 2A, *P* < 0.001), and the result was consistent with that in normal-tumor pairs from the TCGA database (Fig. 2B, *P* = 0.0008). Higher expression of miR-153-5p was observed in the M1 group compared to that in the M0 group (Fig. 2C, *P* = 0.014) and, likewise, miR-153-5p overexpression was closely related to an increased prevalence of M1 (Table 1). The upregulation of miR-153-5p was remarkably associated with the higher TNM (T: the size of tumor; N: lymph nodes involvement; M: distant metastasis) stage (Fig. 2D and Table 1). Besides, the expression levels of miR-153-5p were elevated in patients with lymph node metastasis and higher T stage (Fig. 2E, F). Higher miR-153-5p was found in ccRCC with advanced histological grade (Fig. 2G, *P* = 0.008) and miR-153-5p overexpression showed a remarkable correlation with higher histologic grade (Table 1, *P* = 0.001). Otherwise, ccRCC cell lines increased the expression of miR-153-5p compared to human renal tubular epithelial cells (Fig. 2H).

**Table 1:** Correlations of miR-153-5p and AGO1 mRNA with clinicopathological characteristics in patients with ccRCC.

Parameter	miR-153-5p expression			AGO1 mRNA expression		
	Low	High	*P*	Low	High	*P*
TNM stage						
I + II	161	139	0.045*	140	182	0.001*
III	62	59		72	51	
IV	30	49		52	30	
T stage						
T1 + T2	166	151	0.328	152	188	0.001*
T3 + T4	89	97		113	77	
N stage						
N0	117	103	0.340	114	125	0.501
N1	7	10		9	7	
M stage						
M0	220	196	0.025*	207	233	0.009*
M1	30	47		49	31	
Grade						
G1 + G2	166	151	0.001*	95	146	0.0001*
G3	113	81		108	98	
G4	24	49		55	20	
	^a^Cox coefficient	FDR corrected^a^	*P*^a^			
miR-153-5p	0.245	0.0406	0.0039^*^			
AGO1	−0.231	0.021	0.006^*^			

^a^Multivariate Cox regressions for survival correlations adjusted for sex, age, grade, and histology from OncoLnc.

*P < 0.05.

Next, to investigate the prognostic significance of miR-153-5p, Kaplan–Meier survival analysis was employed according to the dataset from OncoLnc^14^. Results showed that low expression of miR-153-5p contributed to a favorable overall survival in ccRCC (Fig. 2I, *n* = 506, *P* = 0.005). Moreover, multivariate Cox regressions demonstrated that the prognostic impact of miR-153-5p remained significant after adjustment for sex, age, grade, and histology (Table 1, Cox Coefficient = 0.245, *P* = 0.004). These findings indicated that miR-153-5p was an independent prognostic biomarker in ccRCC. What’s more, the Kyoto Encyclopedia of Genes Genomes (KEGG) pathways enrichment analysis and Gene Ontology (GO) analysis were performed to study the miR-153-5p-related signaling. Results showed that miR-153-5p was mainly enriched in DNA replication, proliferation, and other functions, and closely associated with PI3K/Akt signaling pathway (Fig. 2J, K).

### MiR-153-5p promotes the proliferation and metastasis via the PI3K/Akt pathway

**Fig. 3:**
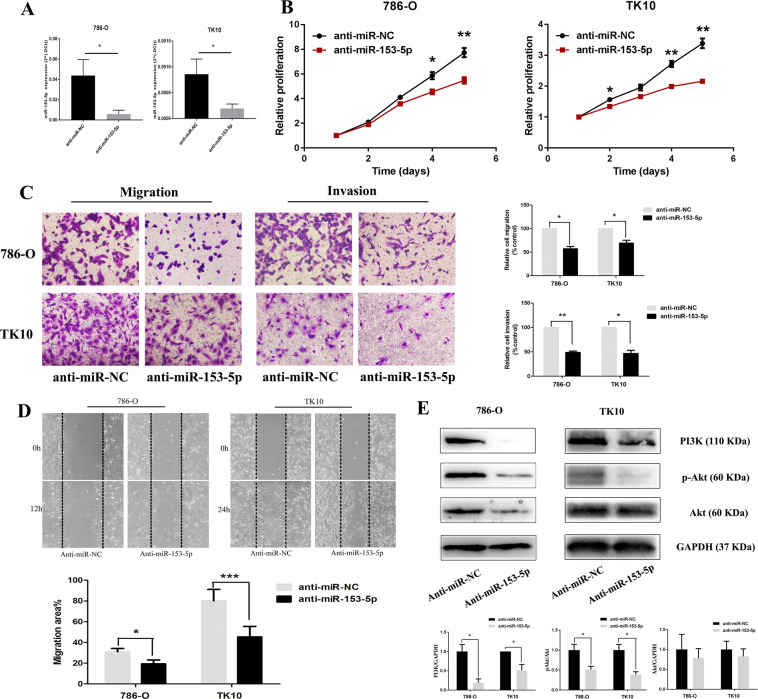
MiR-153-5p knockdown decreased proliferation and metastasis of ccRCC via PI3K/Akt signaling. **A** The expression level of miR-153-5p in 786-O and TK10 after transfection with miR-153-5p inhibitor. **B** The proliferation rate of ccRCC after the miR-153-5p knockdown. **C**, **D** Effect of miR-153-5p depletion on migration (**C**, **D**) and invasion (**C**). **E** Effect of miR-153-5p depletion on PI3K/Akt signaling. All experiments were conducted at least three independent times and all the values were presented as mean ± SEM. *P < 0.05, **P < 0.01, ***P < 0.001.

To explore the role of miR-153-5p on the proliferation and metastasis of ccRCC, we transfected 786-O and TK10 with miR-153-5p inhibitor or NC inhibitor, and performed the Cell counting kit 8 (CCK-8) assay, transwell assay, and wound-healing assay, to examine the effects of miR-153-5p. Results showed that the expression level of miR-153-5p was remarkably decreased after miR-153-5p depletion (Fig. 3A). MiR-153-5p knockdown significantly downregulated cell proliferation rate (Fig. 3B). Transwell assay showed that the migration and invasion ability of ccRCC cells obviously decreased after transfection with miR-153-5p inhibitor (Fig. 3C, D). Likewise, miR-153-5p upregulation with miR-153-5p mimics significantly elevated proliferation, migration, and invasion of ccRCC (Fig. S1). Besides, cell proliferation did not significantly change after transfection with miR-153-5p inhibitor or mimics under serum-free culture conditions (Fig. S2). Thus, the impact of proliferation on invasion and migration could be ruled out. Further investigation of the underlying mechanism of miR-153-5p, we found that miR-153-5p knockdown could significantly reduce the relative expression level of PI3K and p-Akt. Meanwhile, miR-153-5p depletion showed no effect on the relative expression level of Akt. These results indicated that the PI3K/Akt pathway was involved in the stimulative effects of miR-153-5p on proliferation and metastasis (Fig. 3E).

### AGO1 is a direct target of miR-153-5p

**Fig. 4:**
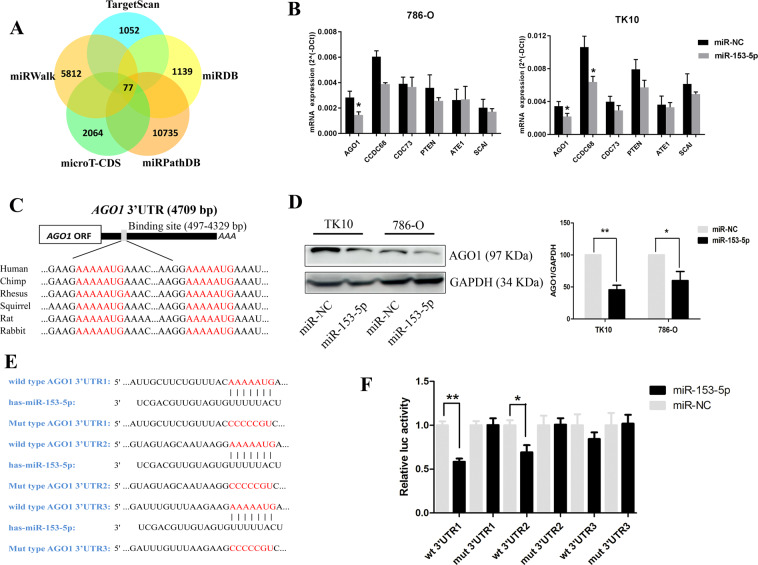
Screen the potential targets of miR-153-5p and AGO1 was identified as its direct target. **A** Schematic illustration displaying the overlapped target genes of miR-153-5p predicted by microT-CDS, TargetScan, miRWalk, miRPathDB, and miRDB. **B** The mRNA expression of the top target genes were tested by qPCR in 786-O and TK10 after transfection with miR-153-5p mimics. **C** Sequence alignment of the predicted miR-153-5p-binding targets in the AGO1 3′-UTR from six organisms. **D** The protein expression of AGO1 was tested in 786-O and TK10 after transfection with miR-153-5p mimics by western blotting. **E** The sequence of binding targets of miR-153-5p in wild-type or mutant AGO1 3′-UTR. **F** After transfection with miR-NC or miR-153-5p in HEK293T cells, the relative luciferase activity of mutant or wild-type AGO1 3′-UTR was detected. All experiments were conducted at least three independent times and all the values were presented as mean ± SEM. *P < 0.05, **P < 0.01.

To investigate the underlying molecular mechanism that was involved in the oncogenic role of miR-153-5p, we screened the potential gene targets of miR-153-5p based on the bioinformatics algorithms of TargetScan, miRWalk, microT-CDS, miRPathDB, and miRDB. A total of 77 genes was overlapped with high stringency (Fig. 4A). We further identified the candidate target genes according to the following criteria: (1) prognosis-related and metastasis-related genes in ccRCC based on GEPIA and LinkedOmics; and (2) had the definite function of tumor suppressor based on literature review. Finally, the top six genes meeting all these above requirements were tested, including *AGO1*, coiled-coil domain containing 68 (*CCDC68*), cell division cycle 73 (*CDC73*), phosphatase and tensin homolog deleted on chromosome ten (*PTEN*), arginyltransferase 1 (*ATE1*), and suppressor of cancer cell invasion (*SCAI*). Next, quantitative PCR (qPCR) was performed to test their mRNA expression. We found AGO1 and CCDC68 were significantly downregulated in 786-O after transfection with miR-153-5p, whereas AGO1 were downregulated in TK10 (Fig. 4B). AGO1 was overlapped in two cell lines and CCDC68 was only significantly downregulated in TK10 cells. However, CCDC68 knockdown did not show an obvious effect on proliferation in ccRCC (Fig. S3). Thus, we speculated that AGO1 may be the potential target of miR-153-5p.

As shown in Fig. 4C, the sequence of the putative bindings are in 497–504, 1623–1630, and 4322–4329 bp of AGO1 3′-UTR. The Homology search demonstrated that putative targets are evolutionarily conserved. The protein expression of AGO1 was remarkably decreased after overexpression of miR-153-5p (Fig. 4D). Subsequently, to explore whether miR-153-5p could directly bind to AGO1 3′-UTR, we cloned the sequence of AGO1 3′-UTR flanked three wild type or three mutant-binding targets of miR-153-5p into the pmiRGLO vector, individually (Fig. 4E). We co-transfected HEK293T cells with miR-NC or miR-153-5p and pmiRGLO-3′-UTR vectors. As illustrated in Fig. 4F, the relative luciferase activities of wild-type AGO1 3′-UTR1 and 3′-UTR2 vectors were significantly reduced after transfection with miR-153-5p. On the contrary, the decrease of luciferase activity was fully abolished when mutant AGO1 3′-UTR1 and 3′-UTR2 were co-transfected with miR-153-5p. However, the luciferase activity of wild-type AGO1 3′-UTR3 did not significantly downregulated when co-transfected with miR-153-5p. Collectively, these results demonstrated that AGO1 serves as a direct target of miR-153-5p.

### AGO1 is associated with favorable clinical features and serves as an independent prognostic biomarker in ccRCC

**Fig. 5:**
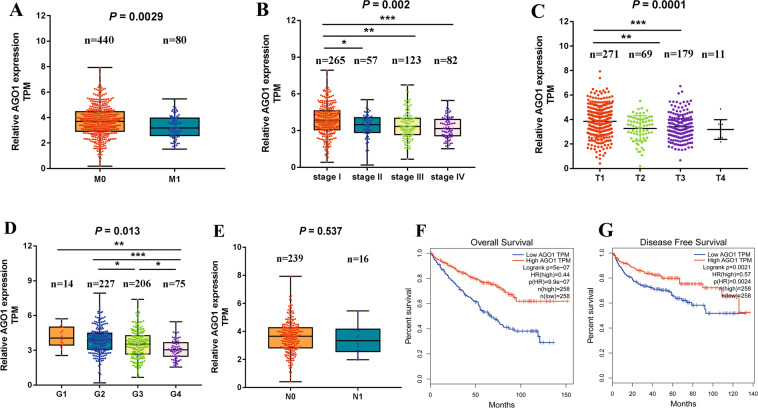
AGO1 is closely related to favorable clinical features and prognosis. **A**–**E** Correlation of AGO1 with clinicopathological characteristics in patients with ccRCC, including metastasis (**A**), TNM stage (**B**), T stage (**C**), histologic grade (**D**), and lymph node metastasis (**E**). Horizontal lines in the boxes in **A**, **B**, **D**, **E** indicated the median value. Boxes spanned the interquartile range. Whiskers extended from the minimum to the maximum. Horizontal lines in **C** represent the mean value. **F**, **G** Kaplan–Meier analysis of overall survival (**F**) and disease-free survival (**G**) regarding AGO1 expression in ccRCC patients. *P < 0.05, **P < 0.01, ***P < 0.001.

To demonstrate the clinical significance of AGO1 in ccRCC, we investigated the correlation of AGO1 expression with clinicopathological features based on the TCGA dataset. The results showed that the expression levels of AGO1 were significantly decreased in patients with M1 than that in patients with M0 (Fig. 5A, *P* = 0.003) and similarly AGO1 overexpression was related to a decreased prevalence of M1 (Table 1, *P* = 0.009). Besides, lower expression of AGO1 was found in higher TNM stage (Fig. 5B, *P* = 0.022), T grade (Fig. 5C, *P* = 0.0001), and histological grade (Fig. 5D, *P* = 0.013). Likewise, AGO1 overexpression had significant correlations with lower TNM stage, T stage, and histological grade (Table 1, all *P* < 0.01). AGO1 expression was decreased in patients with N1; however, no statistical significance was observed (Fig. 5E and Table 1).

As shown in Fig. 5F, G, patients with low AGO1 expression had worse overall survival (hazard ratio (HR) = 0.44, *P* < 0.001) and disease-free survival (HR = 0.57, *P* = 0.002). Moreover, multivariate Cox regressions demonstrated that the prognostic impact of AGO1 remained significant after adjustment for sex, age, grade, and histology (Table 1, Cox coefficient = −0.231, *P* = 0.006), suggesting that AGO1 serves as an independent prognostic biomarker in ccRCC.

### The role of miR-153-5p on tumor progression is mediated via downregulation of AGO1

**Fig. 6:**
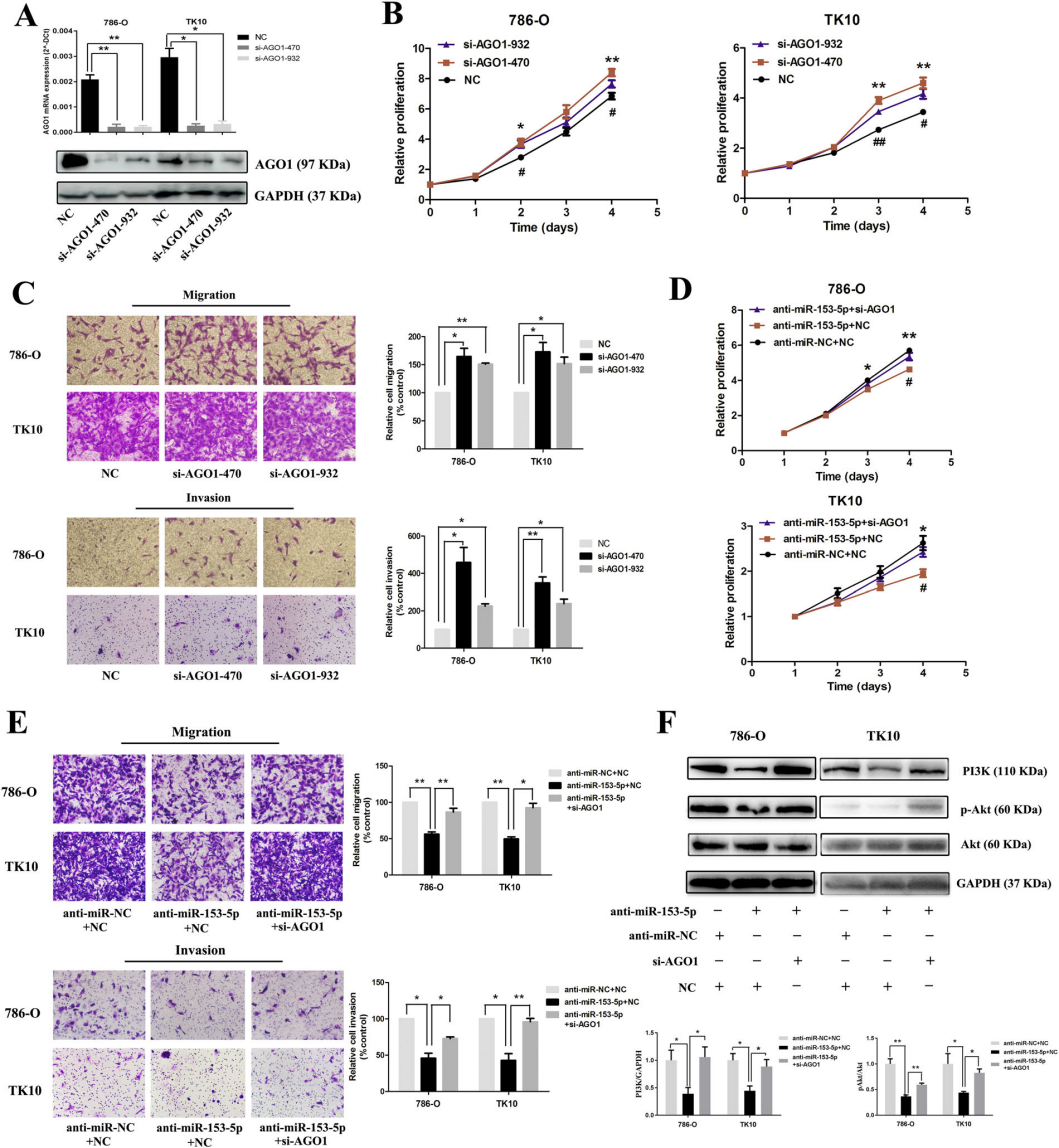
The role of miR-153-5p on tumor progression is mediated via the downregulation of AGO1. **A** The expression level of mRNA and protein of AGO1 in 786-O and TK10 after transfection with siRNA of AGO1. **B** The proliferation rate of ccRCC after AGO1 knockdown. **C** Effect of AGO1 knockdown on migration and invasion. The proliferation (**D**) and metastasis (**E**) of ccRCC after co-transfection with miR-153-5p inhibitor or NC inhibitor with either AGO1 siRNA or NC. **F** The alterations of the PI3K/Akt pathway after co-transfection with miR-153-5p inhibitor or NC inhibitor with either AGO1 siRNA or NC. All experiments were conducted at least three independent times and all the values were presented as mean ± SEM. *P < 0.05, **P < 0.01 (**B** vs. NC; **D** vs. anti-miR-153 + NC); ^#^*P* < 0.05, ^##^*P* < 0.01; **B** vs. si-AGO1-932; **D** vs. anti-miR-153-5p + si-AGO1).

Our results have demonstrated that AGO1 was the direct target of miR-153-5p; we further determined whether AGO1 serves as a functional target gene. Small iterfering RNA (siRNA) of AGO1 (si-AGO1) was employed and the expression of AGO1 was obviously decreased after si-AGO1 treatment (Fig. 6A and Fig. S4). As is shown in Fig. 6B, C, AGO1 knockdown significantly elevated the proliferation, migration, and invasion of ccRCC. Besides, transfection with miR-153-5p inhibitor and/or si-AGO1 showed no obvious effects on the cell proliferation under serum-free culture conditions (Fig. S2). Thus, the impact of proliferation on invasion and migration could be ruled out. Next, we investigated whether the role of miR-153-5p on tumor progression was mediated via downregulation of AGO1. 786-O and TK10 cells were co-transfected with miR-153-5p inhibitor or NC inhibitor and si-AGO1 or NC. Results showed that proliferation, migration, and invasion were remarkably downregulated by the effect of miR-153-5p knockdown, which was significantly reversed by co-transfection with si-AGO1 (Fig. 6D, E). To further explore the underlying mechanism of the AGO1-mediated effect of miR-153-5p, PI3K/Akt pathway was examined when cells were co-transfected with miR-153-5p inhibitor and si-AGO1. Results showed that the PI3K/Akt pathway was obviously suppressed by the effect of miR-153-5p knockdown, which was significantly reversed by co-transfection with si-AGO1 (Fig. 6F and Fig. S4). Taken together, we concluded that AGO1 is a direct functional target of miR-153-5p.

### MiR-153-5p depletion inhibited tumorigenesis in vivo and the miR-153-5p/AGO1 axis serves as a robust prognostic indicator in ccRCC

**Fig. 7:**
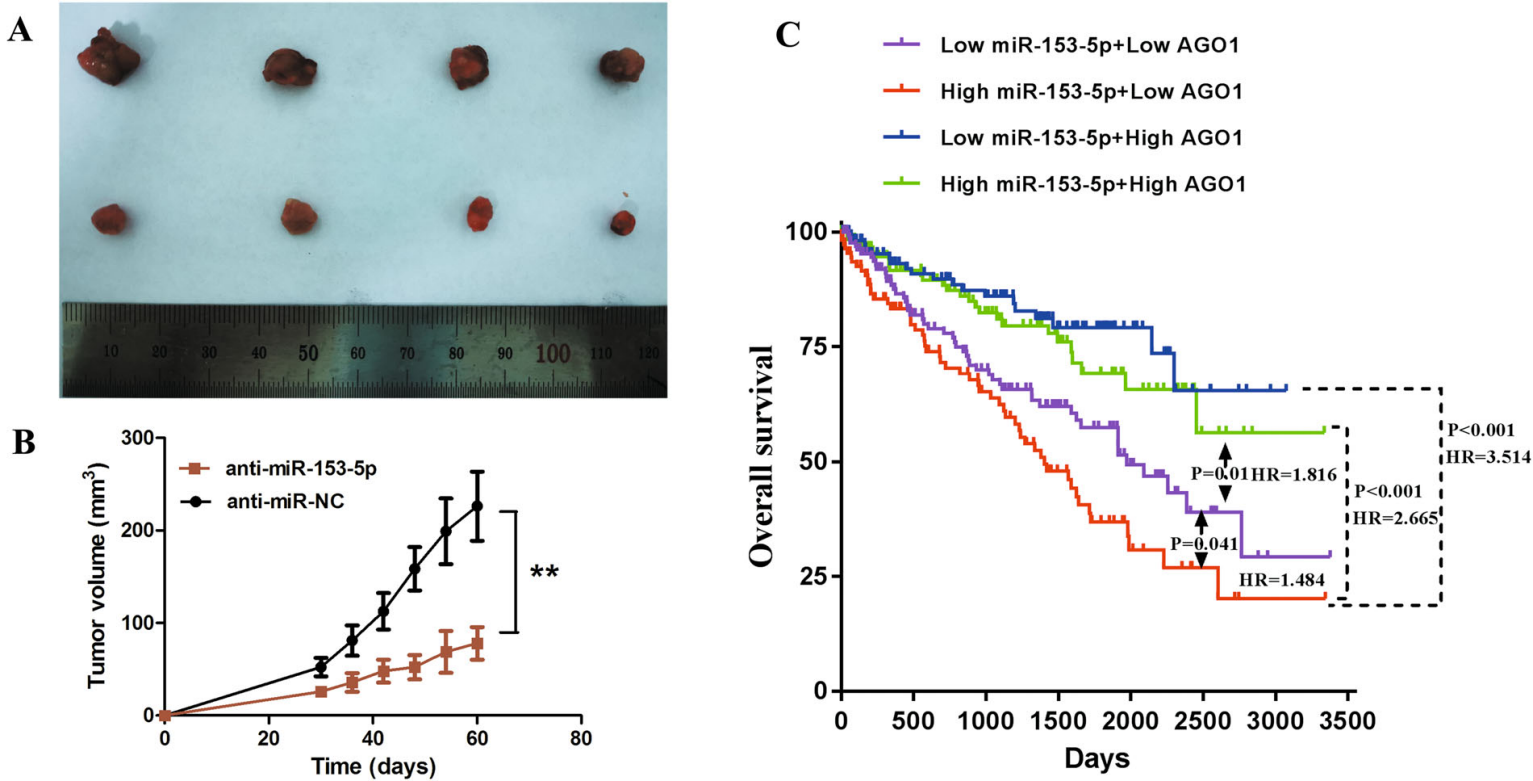
MiR-153-5p knockdown inhibited tumorigenesis in vivo and miR-153-5p/AGO1 axis serves as a robust prognostic indicator in ccRCC. **A**, **B** The tumor growth of 786-O cells infected with LV3-hsa-miR-153-5p inhibitor sponge or LV3-NC. **C** Prognosis analysis stratified by the expression level of miR-153-5p and AGO1 according to the TCGA dataset. All experiments were conducted at least three independent times and all the values were presented as mean ± SEM. **P < 0.01.

We performed in vivo tumor growth assay to validate the effect of miR-153-5p in vivo. 786-O cells infected with LV3-hsa-miR-153-5p inhibitor sponge or LV3-NC were injected subcutaneously into the right armpit of mice. Results showed that the growth speed of tumors in the anti-miR-NC group was significantly faster than that in the anti-miR-153-5p group (Fig. 7A, B). Besides, we conducted a prognosis analysis stratified by the expression level of miR-153-5p and AGO1 according to the TCGA dataset (Fig. 7C). We found that high miR-153-5p + low AGO1 group had the worst prognosis (vs. low miR-153-5p + high AGO1 group: *P* < 0.001, HR = 3.51; vs. high miR-153-5p + high AGO1 group: *P* < 0.001, HR = 2.67; vs. low miR-153-5p + low AGO1 group: *P* = 0.04, HR = 1.48), indicating miR-153-5p/AGO1 axis serves as a robust prognostic indicator in ccRCC.

## Discussion

The clinical intervention of advanced ccRCC remains a challenge because the metastatic diseases commonly become resistant to radical nephrectomy^15^. Due to the resistance to chemoradiotherapy of ccRCC, exploring novel molecular targets are urgently needed^16,17^. MiRNAs have been demonstrated to take part in the regulation of the occurrence and progression of cancers^18,19^ and showed great potential for the prognostic biomarkers^20^. In the present study, we systematically screened differentially expressed miRNAs based on three dimensions of metastasis, prognosis, and differential expression (vs. normal tissue) in ccRCC using bioinformatics algorithms. MiR-153-5p was identified as a candidate oncomiRNA and showed independent prognostic significance in ccRCC. We found miR-153-5p knockdown remarkably inhibited ccRCC cell invasion, migration, and proliferation. Suppression of PI3K/Akt signaling was observed after knockdown of miR-153-5p. Moreover, AGO1 was verified to be a direct target of miR-153-5p and exhibited independent prognostic value. Downregulation of AGO1 significantly promoted the proliferation and metastasis of ccRCC. In the rescue experiments, AGO1 knockdown could partly abolish the inhibition of proliferation and metastasis and reverse the suppression of PI3K/Akt signaling induced by knockdown of miR-153-5p. Furthermore, miR-153-5p combined with AGO1 may be a promising clinical prognostic assessment tool.

Mir-153-2, located on chromosome 7q36.3, was recently demonstrated to be implicated in the pathogenesis of numerous solid tumors or hematological malignancies^11,21–24^. However, the majority of these researches focused on the function of miR-153-3p, the 3′-arm of precursor miR-153, on various cancers including RCC^12,13^. MiR-153-5p, as the homological fragment of miR-153-3p, its related studies are rare and the role of miR-153-5p on ccRCC remains to be explored. Our results showed that miR-153-5p was significantly upregulated in the ccRCC tissues and cell lines. MiR-153-5p depletion remarkably inhibited ccRCC cell proliferation and metastasis. Moreover, miR-153-5p is related to unfavorable clinical features and serves as an independent prognostic biomarker in ccRCC. These findings suggested that miR-153-5p played a crucial role in tumorigenesis and progression.

To further investigate the signaling pathway involved in the oncogenic effects of miR-153-5p, we performed GO and KEGG analysis based on the TCGA dataset. MiR-153-5p was mainly enriched in PI3K/Akt signaling. PI3K/Akt signaling, which plays a pivotal role in the regulation of cell proliferation and metabolism, serves as a crucial growth regulatory pathway in tumors. PI3K/AKT pathway could promote G1/S transition by upregulation of C-myc and Cyclin D1^25^. Apoptosis and autophagy of tumor cells were induced via deactivation of PI3K/AKT^26,27^. Moreover, a previous study has shown that PI3K/AKT was implicated in EMT-related metastasis by overexpression of Slug and Snail meanwhile downregulation of E-cadherin^26^. Clinically, PI3K/AKT signaling is highly activated in ccRCC and several of its targeted drugs have been approved for patients with ccRCC^28^. In the present study, we found that the PI3K/AKT pathway was significantly inhibited after miR-153-5p depletion, indicating that PI3K/AKT signaling may be involved in the oncogenic effect of miR-153-5p.

AGO1, also known as eukaryotic initiation factor 2C1, is one of the members of the AGO protein family (AGO1–AGO4)^29^. AGOs are the core proteins of the miRNA-induced silencing complex^30^ and also exist in all the RISCs^31^, which leads to mRNA degradation and translation inhibition^30,32^. Among the AGO protein family, AGO1 is not only implicated in small RNA-induced gene silencing but also responsible for the unwinding of the miRNA duplexes^33,34^. Several investigations have reported that AGO1 played a role in various cancers. AGO1 was found to suppress tumor angiogenesis and predict a better prognosis via translational inhibition of vascular endothelial growth factor (VEGF)^35^. It has been reported AGO1 acted as a biomarker for colon cancer, melanoma, and muscle-invasive bladder carcinomas^36–38^. However, the role of AGO1 in ccRCC is still unknown. Here we first found AGO1 was a direct target of oncomiRNA miR-153-5p and served as an independent biomarker in ccRCC with an HR value of 0.44. AGO1 remarkably inhibited the proliferation and metastasis of ccRCC, and significantly abolished the oncogenic effects of miR-153-5p. Downregulation of AGO1 could reverse the inhibition of PI3K/Akt signaling caused by miR-153-5p knockdown. These findings demonstrated that AGO1, function as a tumor suppressor gene, was responsible for the oncogenic role of miR-153-5p. Notably, survival analysis showed that ccRCC patients with high miR-153-5p and low AGO1 had the worst prognosis than other patients, indicating that miR-153-5p together with AGO1 may be a promising clinical prognostic assessment tool.

The tumor microenvironment has been demonstrated to be related to metastasis, chemo-resistance, angiogenesis, and immunosuppression of cancer^39,40^. miRNA targeting coding gene plays an important role in the crosstalk of cancer cells with the surrounding microenvironment. Emerging evidence has indicated that several miRNAs, such as miR-221-3p and miR-126, could target VEGF signaling to mediate angiogenesis^40,41^. MiR-770 could transform the M1 phenotype of macrophages to the M2 phenotype by regulating the polarization of macrophages and further induce immunosuppression^39^. Therefore, therapeutic strategies focused on miRNA-related anti-angiogenic therapy and immunotherapy may be promising. Recent studies have indicated that miR-153-5p could directly target Notch^42^ and regulate the angiogenesis in bladder tumors by targeting indoleamine 2,3-dioxygenase 1^42,43^. Thus, miR-153-5p/AGO1 may be a potential therapeutic target for the anti-angiogenic therapy and immunotherapy in ccRCC and remains to be further investigated.

Notably, ccRCC, as an immune sensitive tumor, its dissemination and metastasis may be relevant to the regulation of the immune system. Studies have demonstrated that ccRCC with bone metastasis had a worse prognosis and did not show a good respond to the immunotherapy, which suggested that immune regulation may play a role in the dissemination of ccRCC and have the potential to be a prospective novel therapeutic approach for ccRCC^44,45^.

In summary, we demonstrated that miR-153-5p depletion could significantly inhibit the proliferation and metastasis of ccRCC, which was mediated via PI3K/Akt signaling. AGO1 was identified as a direct functional target of miR-153-5p. Conversely, downregulation of AGO1 promoted the proliferation and metastasis of ccRCC. Moreover, miR-153-5p combined with AGO1 showed more robust prognostic significance in ccRCC. The newly identified miR-153-5p/AGO1 axis may provide promising therapeutic targets and prognostic biomarkers for patients with ccRCC.

## Materials and methods

### Chemicals and reagents

MiR-153-5p inhibitor/mimic, inhibitor/mimic NC, and AGO1 siRNA were purchased from GenePharma (Shanghai, China). The siRNAs were selected by qPCR and the siRNAs with a knockdown efficiency <70% were ruled out. The sequence of siRNA was listed in Table S1. INTERFERin and jetPRIME used as transfection reagents were from Polyplus-transfection® SA (Strasbourg, France). CCK-8 was obtained from Dojindo (Kumamoto, Japan). Dulbecco’s modified Eagle’s medium (DMEM), RPMI 1640 Medium, McCoy’s 5A modified medium, fetal bovine serum (FBS), and 0.25% Trypsin-EDTA were purchased from Gibco (Grand Island, NY, USA). Antibodies to AGO1 (#5053), Akt (#9272 S), p-Akt (#9271 S), PI3K (#13857), and GAPDH (#5174 T) were from Cell Signaling Technology (Beverly, USA). The dual-luciferase expression vector pmirGLO and Dual-Luciferase® Reporter Assay System were obtained from Promega (Madison, WI, USA). All other reagents were from Beyotime (Shanghai, China) and Solarbio (Beijing, China), unless otherwise indicated.

### Cell culture

786-O, Caki-1, HK-2, and HEK293T were obtained from American Type Culture Collection. 786-O and TK10 were cultured in RPMI 1640 medium. Caki-1 cells were maintained in McCoy’s 5A modified medium. HK-2 and HEK293T were cultured in DMEM medium. Cells were cultured in the medium containing 10% FBS, 100 U/mL penicillin, and 100 μg/mL streptomycin, and incubated in an atmosphere of 5% CO_2_ at 37 °C.

### CCK-8 assay

CCK-8 assay was used to detect the cell proliferation rate. 786-O and TK10 cells were seeded in 96-well plates at a density of 4000/well. After incubation overnight, the cells were transfected with miR-153-5p inhibitor/mimic, inhibitor/mimic NC, or AGO1 siRNA. Subsequently, 10 μl CCK-8 reagent was added into each well and incubated for 1 h at 37 °C after transfection for the indicated times. The absorption value at 450 nm was detected on a Microplate Reader (Bio-Rad, Hercules, CA).

### Migration and invasion assays

Migration and invasion assays were conducted using the transwell system (Corning Costar, Lowell, MA, USA) as previously described^46^. In the migration assay, 4 × 10^4^ 786-O cells or 1 × 10^5^ TK10 cells were suspended in 200 μl of serum-free medium and added into the upper chamber. Subsequently, 600 μl complete medium was added to the lower chamber. After incubation for 24 or 36 h, the cells that migrated through the membrane were fixed for 15 min with 4% paraformaldehyde and then incubated in 0.1% crystal violet for 30 min. For the invasion assay, the procedures were exhibited in the same way as mentioned above except the membranes were coated with Matrigel (BD Bioscience, USA). The cell count was quantified in three representative fields in each independent experiment.

### Wound-healing assay

The cells were seeded into the six-well plates to reach 100% confluence after transfection. Horizontal lines were drawn on the back of the plate; prior to that, a linear scratch was created on a six-well plate by 200 μl pipette tips and then the detached cells were removed by phosphate-buffered saline (PBS). Subsequently, the pictures of scratch were taken under the microscope for the indicated times at magnification of ×200 and the images were analyzed by ImageJ.

### RNA isolation and qPCR assay

Total RNA was extracted from cells or tissues with TRIzol reagents (Invitrogen, Carlsbad, CA, USA) as the instruction of the manufacturer’s protocol. For mRNA, the Prime-Script RT reagent kit (TaKaRa, Shiga, Japan) was employed to synthesize cDNA from RNA and real-time PCR was conducted with SYBR Premix Ex Taq (TaKaRa) using CFX96 Real-time PCR Detection System. miRNA was reverse transcribed by miRNA First-Strand Synthesis Kit (Sparkjade, Qingdao, China) and reverse-transcription PCR was performed using miRNA Real-time PCR Kit (Sparkjade). Primers of several genes were designed including miR-153-5p, AGO1, β-actin, CCDC68, CDC73, PTEN, ATE1, and SCAI. Primer information was listed in Supplementary Table S1.

### Dua-luciferase reporter assay

The 3′-UTR of AGO1 carrying different putative miR-153-5p-binding sites were amplified and then cloned into pmirGLO. The miR-153-5p complementary sites with the sequence 5′-AAAAATGA-3′ in AGO1 3′-UTR were singly mutated to eliminate the complementarity to miR-153-5p as described previously^47^. Cells were seeded in 24-well plates and then co-transfected with wild-type or mutated AGO1 3′-UTR constructs, and miR-153-5p mimics or NC. Forty-eight hours after transfection, the cells were harvested, and the luciferase activity was measured using the Dual-Luciferase® Reporter Assay System. Normalized luciferase activity was calculated as luciferase activity against *Renilla* luciferase activity.

### Western blot analysis

Western blotting was performed as described previously^48,49^. Cells were lysed using RIPA buffer with Phenylmethanesulfonyl fluoride and phosphatase inhibitors, and total proteins were extracted after transfection. The Protein was separated by 10% SDS-polyacrylamide gel electrophoresis and then electro-transferred to polyvinylidene difluoride membrane. The membranes were then collected and blocked by the blocking buffer containing 5% nonfat milk for 1 h. Subsequently, the bands were incubated with primary antibodies overnight at 4 °C and then with secondary antibodies for 1 h at room temperature. The bands were detected by the Pro-lighting HRP regent and semi-quantitatively analyzed by ImageJ.

### In vivo tumor growth assay

Four- to 5-week-old NOD SCID male mice were purchased from Beijing Vital River Laboratory Animal Technology Co. Ltd (Beijing, China). Eight mice were randomly divided into two groups (*n* = 4). 786-O cells were infected with LV3-hsa-miR-153-5p inhibitor sponge or LV3-NC (GenePharma, Shanghai, China). 786-O cells with two different transfections (each 5 × 10^6^ cell) were suspended in 200 μl PBS and injected subcutaneously into the right armpit of two groups of mice, respectively. Tumor size was monitored every 6 days and the volumes were measured using the following formula: volume = 1/2 length × width^2^. The mice were killed and tumors were collected 2 months post injection. All animal procedures were approved by the Ethical Committee of Shandong University.

### Bioinformatic analysis

TCGA data of ccRCC, including RNA sequencing and clinical data for 522 tumors and 72 normal tissues, were downloaded from the Genomic Data Commons Data Portal^50^. Differential expression analysis and volcano plots graphing were performed using R programming language, which is a software environment for graphics and statistical computing. KEGG pathways enrichment analysis and GO enrichment analysis was performed to investigate the miR-153-5p-related signaling. GEPIA^51^, LinkedOmics^52^, and OncoLnc^14^ websites were employed to conduct the expression analysis and survival analysis of miR-153-5p or AGO1.

### Statistical analysis

Statistical differences between two groups were assessed using Student’s *t*-test and one-way analysis of variance was employed to identify the statistical differences among multiple groups. For the data not meet normal distribution, the Mann–Whitney test and Kruskal–Wallis test were used, respectively. Pearson’s *χ*^2^-test was used to compare clinicopathological features between groups when the data conformed to normal distribution. Kaplan–Meier method was employed to plot survival curves by the log-rank test. Differences with a two-tailed *P* < 0.05 were considered significant. GraphPad Prism Software was employed for statistical analysis.

## Supplementary information

Table S1

Figure S1

Figure S2

Figure S3

Figure S4

supplementary figure and table legends
