# Supplementary material for: MicroRNA-153-5p promotes the proliferation and metastasis of renal cell carcinoma via direct targeting of AGO1
Source: Cell Death Dis. 2021 Jan 4;12(1):33. doi: 10.1038/s41419-020-03306-y (PMC7791042; doi:10.1038/s41419-020-03306-y)
Supplement: Supplementary file 1 — Table S1 [file 41419_2020_3306_MOESM1_ESM.docx]

**Supplementary Table S1**: Primers used for qPCR and sequence of siRNA

| qPCR | miR-153-5p | F | GCGCGTCATTTTTGTGETGTT |
| --- | --- | --- | --- |
|  |  | RT Primer | GTCGTATCCAGTGCAGGGTCCGAGGTA  TTCGCACTGGATACGACAGCTGC |
|  | AGO1 | F | CCGGTGTATGCTGAGGTGAA |
|  |  | R | TGTTGAAAAACGGCAGAGCG |
|  | CCDC68 | F | AGTGAGAAAGAAGCAGGAGGAC |
|  |  | R | TGTAGCACTGGATTTGAGTTGC |
|  | CDC73 | F | GGACTGGAAAGGAAGGCCAA |
|  |  | R | ATACTTGCCGATGTTGACGC |
|  | PTEN | F | CTCAGCCGTTACCTGTGTGT |
|  |  | R | AGGTTTCCTCTGGTCCTGGT |
|  | ATE1 | F | TTTGCTGTGCCCTGAGACAT |
|  |  | R | CCGTACTGCGATCCTCATCC |
|  | SCAI | F | ATCGCCTTGCTGAAACAGGA |
|  |  | R | AATGAGTGCGTCAGCCAGAG |
|  | β-actin | F | CATGTACGTTGCTATCCAGGC |
|  |  | R | CTCCTTAATGTCACGCACGAT |
| siRNAs | AGO1-homo-470 | | GCCUCAGAUCUUUGGUGAUTT  AUCACCAAAGAUCUGAGGCTT |
|  | AGO1-homo-932 | | GCUGGACAUCAGGAACAUATT  UAUGUUCCUGAUGUCCAGCTT |
|  | CCDC68-homo-432 | | GCACUGUGGAAACCUUCAATT  UUGAAGGUUUCCACAGUGCTT |
|  | CCDC68-homo-945 | | CCAGGAGCAGAUCUCUCAUTT  AUGAGAGAUCUGCUCCUGGTT |
