# Supplementary figures and images for: MicroRNA-153-5p promotes the proliferation and metastasis of renal cell carcinoma via direct targeting of AGO1
Source: Cell Death Dis. 2021 Jan 4;12(1):33. doi: 10.1038/s41419-020-03306-y (PMC7791042; doi:10.1038/s41419-020-03306-y)

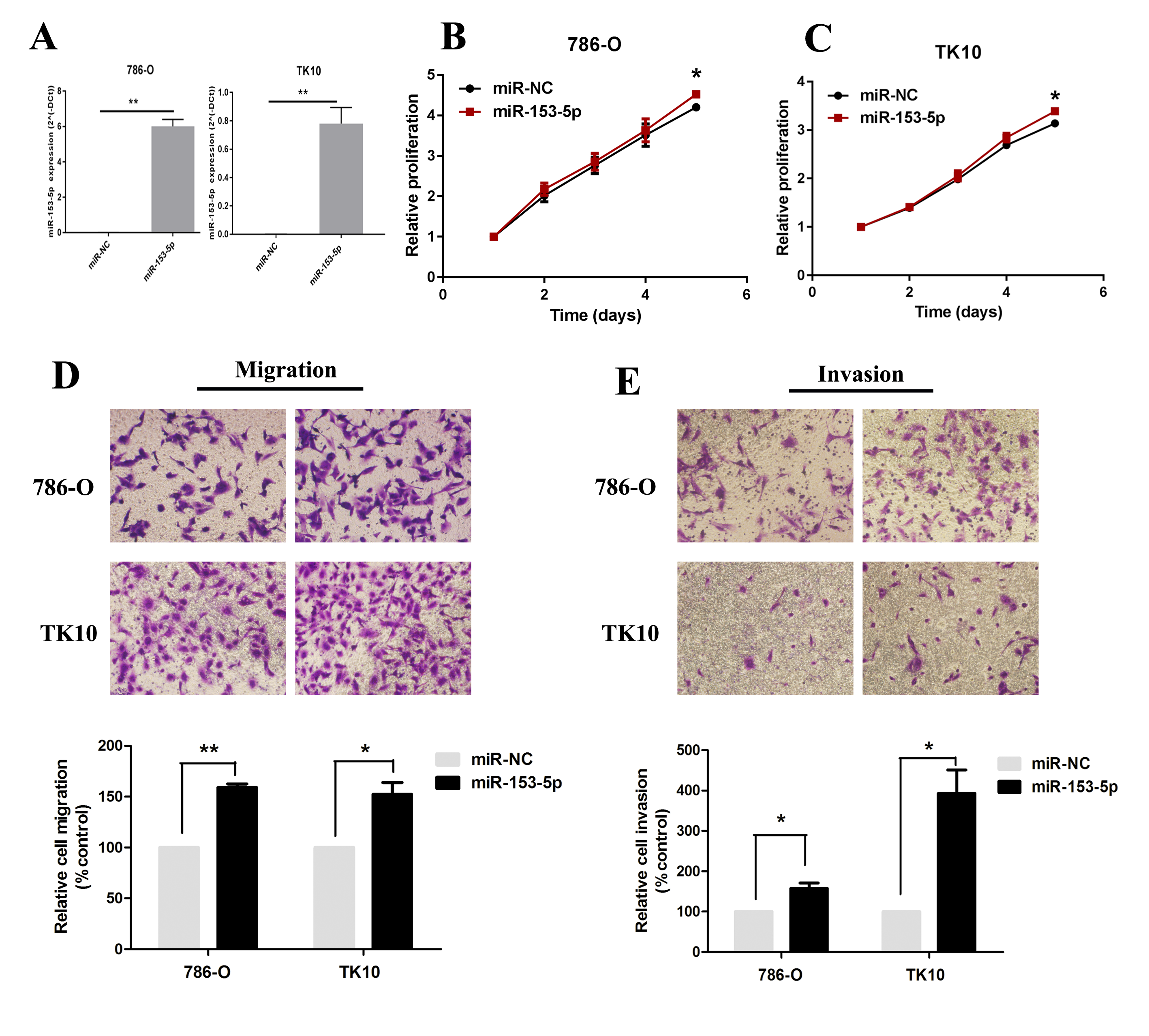

Supplement: Supplementary file 2 — Figure S1 [file 41419_2020_3306_MOESM2_ESM.tif]

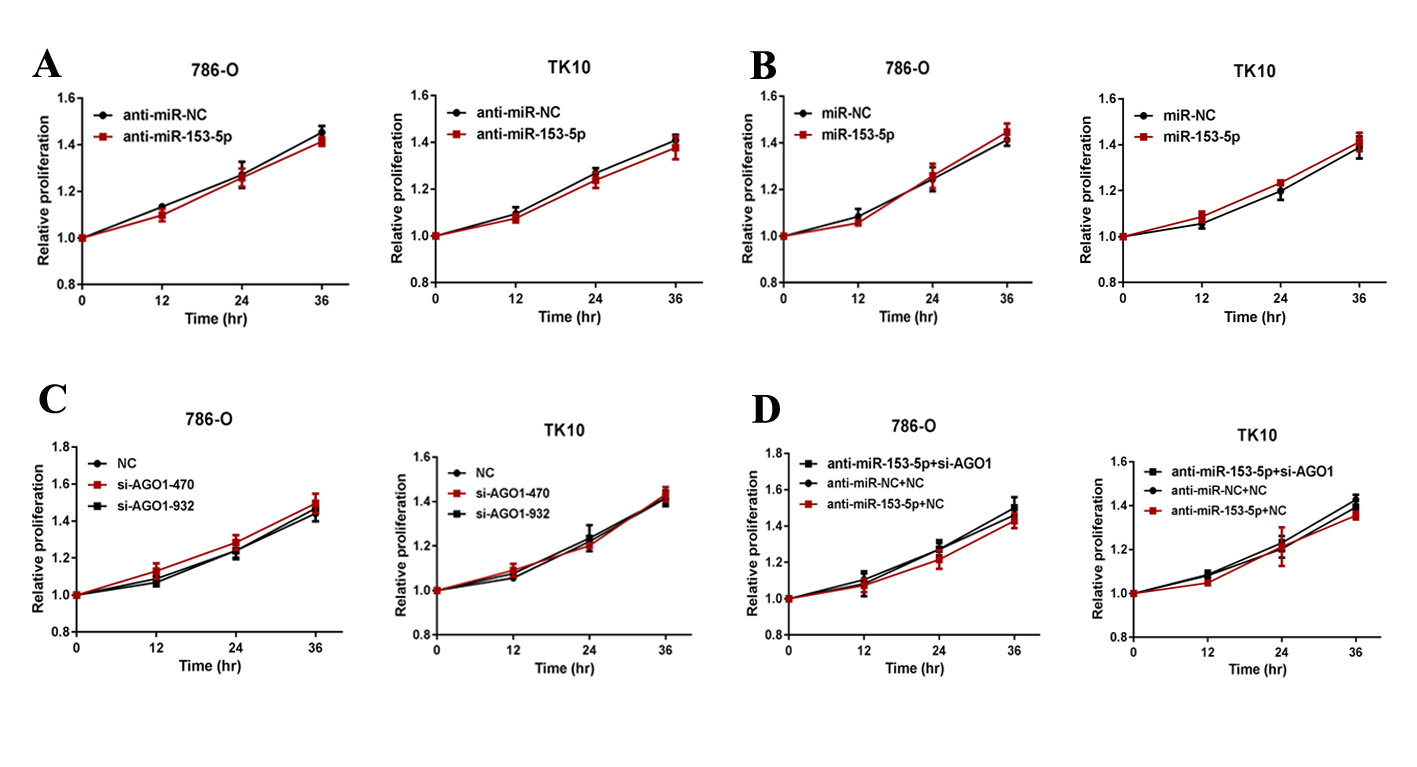

Supplement: Supplementary file 3 — Figure S2 [file 41419_2020_3306_MOESM3_ESM.tif]

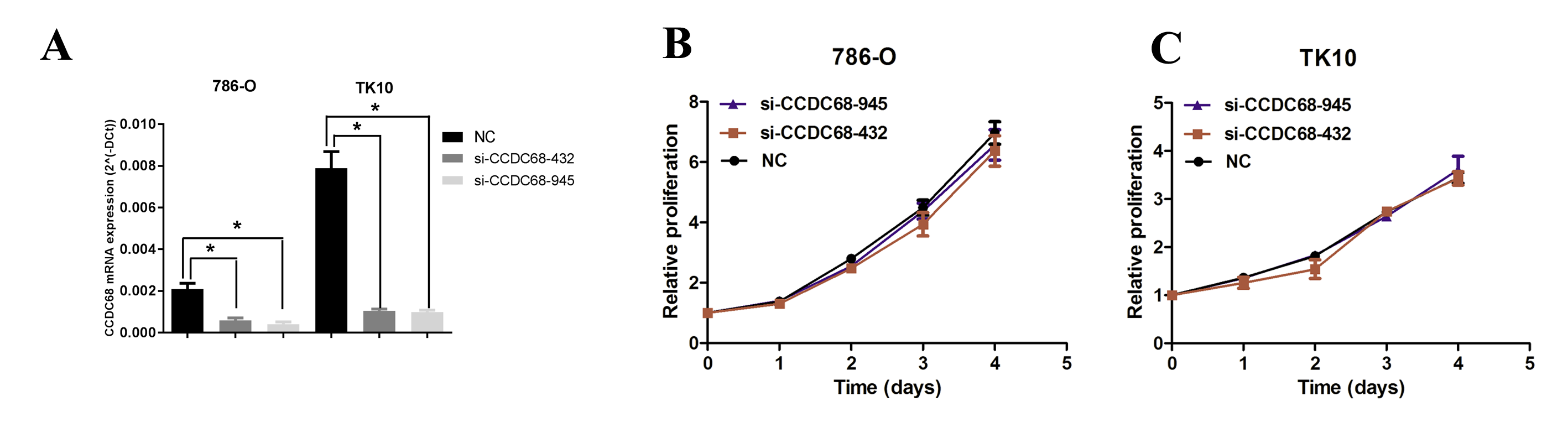

Supplement: Supplementary file 4 — Figure S3 [file 41419_2020_3306_MOESM4_ESM.tif]

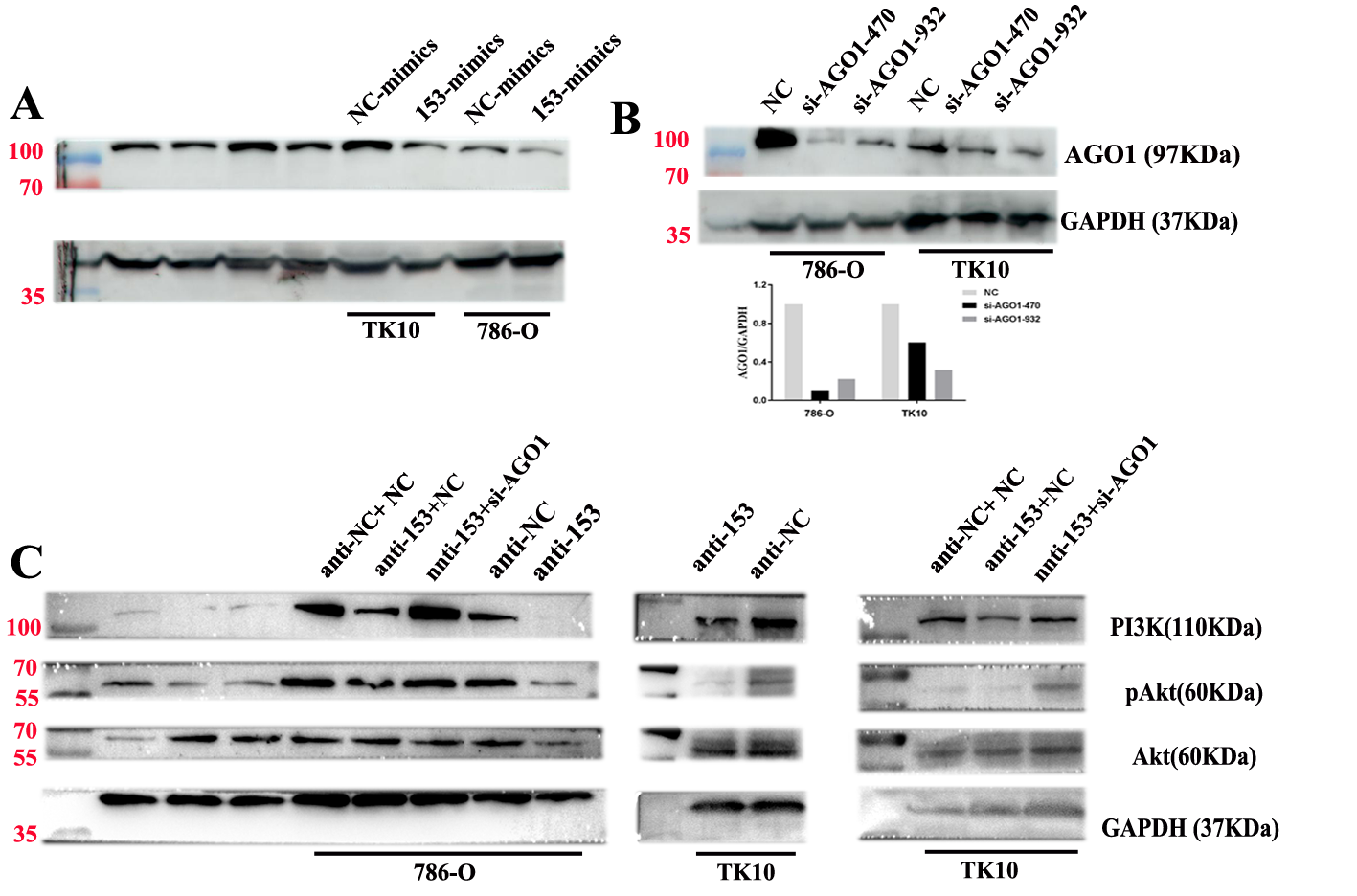

Supplement: Supplementary file 5 — Figure S4 [file 41419_2020_3306_MOESM5_ESM.tif]
