# Supplementary material for: MicroRNA-153-5p promotes the proliferation and metastasis of renal cell carcinoma via direct targeting of AGO1
Source: Cell Death Dis. 2021 Jan 4;12(1):33. doi: 10.1038/s41419-020-03306-y (PMC7791042; doi:10.1038/s41419-020-03306-y)
Supplement: Supplementary file 6 — supplementary figure and table legends [file 41419_2020_3306_MOESM6_ESM.docx]

Fig. S1 MiR-153-5p overexpression increased proliferation and metastasis of ccRCC. **a** The expression level of miR-153-5p after transfection with miR-153-5p mimics. **b**, **c** The proliferation rate of 786-O and TK10 after miR-153-5p overexpression. **d**, **e** Effect of miR-153-5p overexpression on migration **d** and invasion **e**. All experiments were conducted at least three independent times and all the values were presented as mean ± SEM. **P* < 0.05, ***P* < 0.01.

Fig. S2: The effects of transfection with miR-153-5p mimics, miR-153-5p inhibitor, and si-AGO1 on the cell proliferation under serum-free culture condition.

After transfections, 786-O and TK10 cells were seeded in 96-wells plates at a density of 4000/well and then cultured in medium without serum. CCK8 assay was performed at 12h, 24h and 36h after seeding. a, b The proliferation rate of 786-O and TK10 after transfection with miR-153-5p inhibitor a or miR-153-5p mimics b. c The proliferation rate of ccRCC after transfection with si-AGO1. d The proliferation rate of ccRCC after co-transfection with miR-153-5p inhibitor and si-AGO1. All experiments were conducted at least three independent times and and all the values were presented as mean ± SEM.

Fig. S3: The effect of CCDC68 on the proliferation of ccRCC. **a** The expression level of CCDC68 mRNA after transfection with CCDC68 siRNA. **b**, **c** The proliferation rate of 786-O **b** and TK10 **c** after CCDC68 depletion. All experiments were conducted at least three independent times and all the values were presented as mean ± SEM. **P* < 0.01.

Fig. S4: The bands of western blot with molecular markers.

**a** The expression level of AGO1 after transfection with miR-153-5p mimics both in 786-O and TK10. **b** The expression level of AGO1 after transfection with si-AGO1 both in 786-O and TK10. **c** The expression of PI3K/Akt signaling after transfection with miR-153-5p inhibitor or co-transfection with miR-153-5p inhibitor and si-AGO1 both in 786-O and TK10.

Table S1: Primers used for qPCR and sequence of siRNA
